# Toward systematic integration between self-determination theory and motivational interviewing as examples of top-down and bottom-up intervention development: autonomy or volition as a fundamental theoretical principle

**DOI:** 10.1186/1479-5868-9-23

**Published:** 2012-03-02

**Authors:** Maarten Vansteenkiste, Geoffrey C Williams, Ken Resnicow

**Affiliations:** 1 Department of Psychology, Ghent University, Ghent, Belgium; 2 Departments of Medicine and of Clinical and Social Psychology, University of Rochester, Rochester, USA; 3 School of Public Health, University of Michigan, Michigan, USA

## Abstract

Clinical interventions can be developed through two distinct pathways. In the first, which we call top-down, a well-articulated theory drives the development of the intervention, whereas in the case of a bottom-up approach, clinical experience, more so than a dedicated theoretical perspective, drives the intervention. Using this dialectic, this paper discusses Self-Determination Theory (SDT) [1,2] and Motivational Interviewing (MI) [3] as prototypical examples of a top-down and bottom-up approaches, respectively. We sketch the different starting points, foci and developmental processes of SDT and MI, but equally note the complementary character and the potential for systematic integration between both approaches. Nevertheless, for a deeper integration to take place, we contend that MI researchers might want to embrace autonomy as a fundamental basic process underlying therapeutic change and we discuss the advantages of doing so.

## 

An important goal for public health and behavioral medicine researchers and practitioners is to develop effective behavior change interventions [4]. A key component of inducing long term behavior change is building sustained motivation, such that people not only engage in the health behaviors during the initial intervention period but afterwards as well.

Researchers and practitioners may follow quite different trajectories in developing effective motivational interventions [5]. In the top-down approach, theoretically oriented researchers generate a theory for understanding behavior, emotion and/or cognition, and then construct an intervention rooted in their theoretical perspective. In this approach, theory drives intervention. Alternatively, in a bottom-up approach, clinical intuition and clinical experience drive the intervention more than any particular theoretical framework, although developers of a practice driven intervention may turn to existing theories to understand how and why their intuitive interventions work.

Self-Determination Theory (SDT) [1,2] and the interventions it has spawned, might be considered as a prototypical example of the top-down approach, as it represents a broad-band theory on personality and human functioning in social context, whereas Motivational Interviewing [3,6] could be considered an example of the bottom-up approach, as it primarily grew out of clinical practice and necessity rather than out of any particular theory. Indeed, as noted by Miller and Rollnick [7], "MI was not a product of rationale deduction from [such] theories. Rather it represented a clinical method, and later a growing body of empirical findings, in need of theoretical explanation" (p. 134).

Below we compare and contrast these two approaches; although we focus on SDT and MI as examples of the two developmental trajectories, we propose some general guidelines that might inform both research and clinical practice beyond these two examples. Several scholars have argued that SDT can be used as a de facto framework for understanding why MI works [8,9]. Nevertheless, we believe that the potential for systematic integration of these two approaches has not been fully exploited. By describing the different starting points and different developmental histories of SDT and MI as prototypical examples of top-down and bottom-up research, we try to provide the basis for how the two can be more clearly understood with respect to each other. We hope this will promote greater integration and differentiation based on areas of overlap and uniqueness, and further the science of motivation, and the effectiveness of clinical interventions. In particular, we argue that MI adherents might benefit from more fully embracing the theoretical principle of autonomy, as emphasized in SDT.

### SDT's Top-Down Approach

Lewin's [10] statement that "there is nothing more practical than a good theory" might be used to characterize the top-down approach. It is assumed that the development of an internally coherent theory is important, not just for its theoretical elegance, but also because it allows for hypothesis driven research to test mechanisms underlying behavior change. Once these pathways and processes are identified, interventions can be developed. A key advantage in the top-down approach is that it lends itself to understanding how and why an intervention works, not only if.

The starting point of top-down development often involves a clear articulation of a *meta-theoretical *foundation, that is, a view on human kind that serves as a philosophical underpinning of one's theorizing. To illustrate, within SDT [11], an organismic viewpoint is embraced. It is assumed that individuals have a natural inclination to be active and to move their lives in desired and specific directions rather than being passive and completely subjected to environmental forces that push them around (e.g., through the use of punishments and rewards). Through this activity, people would move towards increased integration, growth and wellness given sufficient supportive circumstances. As growth-oriented organisms, individuals stand in a continual interface with the social environment, which can either support and facilitate the unfolding of this natural growth process or block it by frustrating people's basic psychological needs [1] for autonomy (i.e., experiencing a sense of psychological freedom), competence (i.e., experiencing a sense of effectance) and relatedness (i.e., experiencing a sense of intimacy and connectedness).

The articulation of this meta-theory provides the foundation for developing a formal theory in a systematic, research-driven way within the top-down approach. New ideas get naturally and steadily integrated into the theory following sufficient empirical support, which helps the theory to maintain internal consistency. To use a metaphor, theory development with the top-down approach resembles the construction of a *puzzle *[12]. Over the years, new pieces of the puzzle get only added once their fit is determined. Such evolution seems to typify the development of SDT, whose five mini-theories (i.e., cognitive evaluation theory, organismic integration theory, causality orientation theory, basic psychological need theory, and goal content theory) have been gradually developed over the past four decades [12]. As the theoretical puzzle is growing, over time, the theory might be perceived as highly complex. However, when the internal logic of the theory is fully understood, SDT is characterized by a sense of parsimony and elegance, as a minimum set of concepts is used to explain a wide variety of phenomena across age groups, life domains, and cultures. This is because new pieces are added to the theory when (a) the new piece represents a "compelling theoretical necessity", that is, when a clear and meaningful argument can be provided for the enlargement of the theory, and (b) when sufficient empirical support is gathered for the theoretical claim. To illustrate, whereas the satisfaction of the basic needs for autonomy and competence was considered critical for the maintenance and development of intrinsic motivation [13], it is only when the process of internalization - that is, the personal endorsement of externally offered norms, values and regulations - was studied that the basic need for relatedness received a more prominent place [1]. Indeed, people often engage in enjoyable and intrinsically motivating activities by themselves (e.g., reading), that is, without significant others being present to support them, while the development of satisfying relations is critical for individuals to fully endorse and willingly subscribe (i.e., internalize) to norms and behaviors they don't particularly find interesting, and, as a result would not spontaneously engage in [1,12]. Because most health related behaviors (e.g., stopping smoking, getting a mammogram to screen for cancer) fall into this category, SDT-based interventions involve the facilitation of internalization through the support of relatedness in addition to autonomy and competence. Several studies have confirmed the importance of relatedness satisfaction for the internalization process [1].

Due to the focus on the explanatory processes underlying change in the top-down approach, it becomes possible to *a priori *predict specific social-contextual variables that promote change. Within SDT, for instance, counselors are encouraged to support clients' basic psychological needs as much as possible as the experience of need satisfaction leads to a greater integration and anchoring of change into one's life style and values, resulting in maintained change [14]. More specifically, the concept of basic need satisfaction allows delineating specific motivating counseling techniques (e.g., shared agenda setting, choice etc.) and orientations (e.g., empathic style, unconditional regard) (see Patrick & Williams this issue for a more elaborated discussion). Dozens of self-report and experimental studies within SDT have studied such specific need-supportive practices. To illustrate, choice provision is considered as a key strategy to support participants' volition. Such need-supportive strategies were first studied in the laboratory where typically university students would serve as study participants [15,16]. Based on these basic lab-based findings, studies were set up to test the role of choice across several real world domains where it was found to yield manifold advantages, including the facilitation of heart disease management among senior women (> 60 years; [17]), the reduction of drop-out rates among eating disorder clients [18], the promotion of well-being among elderly who stay in nursing homes [19], and the increase of vitality among students in PE classes [20,21]. Thus, real-life experiments complemented the initial laboratory studies, thereby increasing the ecological validity of the theory.

Based on the examination of different experimentally isolated need-supportive components (e.g., choice, rationale provision, etc.) and the development of reliable measures to assess theoretical constructs, SDT scholars more recently proceeded to set up controlled clinical trials in which a theory-driven intervention is tested against a control group. An SDT driven intervention was composed by combining previously isolated need-supportive components. For instance, while rationale provision [22,23], empathy [24], and autonomy-supportive vs. controlling language [25,26] had been examined in isolation in experimental studies, these and other need-supportive facets were taken together to create a need-supportive intervention. Over the past several years, an increasing number of SDT based controlled clinical trials in a wide variety of domains, including physical activity ([27], Fortier, Duda, Guerin, & Teixeira, this issue), tobacco dependence [28], dental care [29] and weight loss [30] have been conducted [31]. The advantage of such controlled clinical trials is that they further confirmed the ecological (or external) validity of SDT in the real world, and established the efficacy of the interventions.

However, this intervention research is still in its infancy phase and, hence, various issues deserve further attention. First, because various need-supportive components are simultaneously manipulated in controlled clinical trials, it remains unclear which of the components are driving the observed effects, an observation that also applies to MI. Future studies may want to isolate these various need-supportive components, as done by Deci, Eghrari, Patrick, and Leone [24] and Sheldon and Filak [32] in laboratory studies as to examine main and interaction effects between those isolated components. This would help to answer the question whether the whole is more than the sum of various need-supportive components; for instance, within SDT, it is maintained that for competence support to yield a truly vitalizing effect such competence support is best provided in an autonomy-supportive rather than controlling way [25], thus suggesting that positive feedback and autonomy-supportive language can best be simultaneously provided. Second, the efficacy and effectiveness of a SDT intervention relative to other psychotherapeutic approaches has not extensively been examined yet, as most of the available controlled clinical trials contrast the experimental group with a no-treatment control group.

Third, although various key ingredients of a need-supportive intervention have now been identified (see Patrick & Williams, this issue), this list remains open for additions and refinements. This is because within a top-down approach as SDT cross-fertilization between basic laboratory studies and (experimental) field studies is pursued. Through laboratory studies, the effects of isolated need-supportive components can be examined, which then might provide the basis for refining the intervention approach. To illustrate, although choice has on average a motivating and vitalizing impact, laboratory studies [33] have demonstrated that choice can be provided in a controlling rather than an informational way such that participants do not experience a *subjective *sense of choice, willingness, and psychological freedom in spite of being offered an *objective *array of options to choose from. Also, an overload of options to choose from has been found to undermine the motivating impact of choice as people are less likely to feel effective to make an accurate choice in such a case [34].

Such research is likely to have important counseling implications: although a clinician might at the surface offer a client who hesitates to stop therapy the choice to do so, the client might feel pressured to continue the counseling if this choice is provided in a controlling and anxiety-provoking way (e.g., "it is up to you to choose to leave treatment or to stay but I can tell you that many clients who leave treatment fail to make it on their own"). Regarding the issue of option overload, although a doctor might provide a number of options to a client how to deal with his situation, the client, already anxious about his health condition, might feel overwhelmed and not have the energy to process all of the information independently. In such a situation, the autonomy-enhancing effect of choice would be diminished by its anxiety-inducing and competence-thwarting effects. What is important in such a situation is not to take away the choice, but to structure the situation such that the provided options become manageable for the client so that he ultimately feels competent to choose and fully endorse one option.

Such basic experimental research is considered fundamental within SDT as it helps to understand how social-contextual factors can simultaneously affect the satisfaction of multiple basic needs. This basic research provides more detailed insight in the need-supportive practices that are characteristic for a SDT intervention and the way how they need to be introduced during counseling to be optimally motivating.

### MI's Bottom-up Approach

Within a bottom-up approach, the starting point for creating interventions is quite different. First, interventions may be driven by increased *prevalence *or *incidence *of particular problems or pathologies. For instance, an increasing number of individuals have developed a sedentary lifestyle and currently suffer from overweight and obesity [35], while a substantial percentage of individuals display low body satisfaction [36], anorexia nervosa or bulimia [37] and roughly 20% of U.S. adults smoke [38]. Also, the personal, social and societal *costs *associated with a particular pathology might prompt a call for action. A second driver of bottom-up can be intervention failure of current approaches or the relative advantage of new approaches, which then form the impetus for setting up interventions.

Within the bottom-up approach, developers are very open to modify their model as they don't have a strong theoretical foundation. More specifically, bottom-up intervention development can involve three different approaches to incorporating theory. First, developers might adopt an *a-theoretical *approach, that is, they do not concern themselves with why their intervention may work instead they may focus solely on whether the intervention works. Second, developers can incorporate theory in a *discovery*-based fashion, that is, they build their model as they discover new findings and as their experience grows. Third, developers can seek inspiration in existing theories to explain why their interventions are effective. Different portions of multiple existing theories are then *eclectically combined *into a new model. We use MI to illustrate the latter two approaches to theory: MI's development seems to be discovery-based and eclectic at the same time, thereby drawing on multiple theories, including SDT.

The term *model *is often used by scholars and clinicians working from the MI perspective [6], which reflects the fact that MI primarily is a clinical method and was not developed from any dedicated theory. Indeed, the development of MI can, to some extent, be described as a discovery trip. For instance, MI evolved from the experience that confronting alcoholics was counterproductive, while being empathic and understanding facilitated abstinence [39,40]. Those initial observations resulted in the first steps in developing a motivational model to promote internally driven change [41]. Over the past two decades [6], the key principles characterizing MI (being empathic, rolling with resistance, promoting change talk, and enhancing self-efficacy) as well as the specific communication techniques that help to put those principles in practice have been extensively described and elaborated (see Resnicow, this issue).

Further, dozens of randomized controlled trials in very diverse populations have demonstrated the effectiveness of MI as a clinical method, both in contrast to 'weak' comparison groups (e.g., waitlist or no treatment groups) and, in some cases, even compared to active treatments rooted in a psychotherapeutic approach (see the meta-analyses of [42-46]). Whereas MI was initially tested in the fields of alcohol and drug use, the range of domains in which it has been tested has been dramatically extended to include tobacco dependence, physical activity, diabetes, dieting, medication adherence, HIV and STI behaviors, heart disease risk reduction, sexual offending, and weight loss. Furthermore, MI has been used as a prelude to various other psychotherapeutic approaches (e.g., CBT; [47,48]). Nonetheless, MI's bottom-up approach seems to have limited its application to health-related or clinical situations, while SDT, as a macro-theory of motivation, has been tested in other life domains as well (e.g., education, sport, parenting, work, etc.).

Despite the substantial evidence for effectiveness in the health care and clinical domain, it is only recently that MI scholars have started to pay attention to 'how and why MI works' [6]. The post-hoc articulation of these underlying processes might be more characteristic of models that are developed bottom-up, because such models don't start from a strong theoretical foundation. Since Miller and Rollnick's call for process oriented research, the mechanisms that might be responsible for the observed positive effects of MI have received more empirical attention [49,50]. In our view, these mechanisms were, just as the counseling approach itself, discovered posteriori rather than being a priori theorized. Having noticed that a MI intervention among drug abuse users failed to yield desire positive effects [51], Amrhein, a psycholinguist collaborating with Miller, deconstructed and analyzed the naturally occurring change and resistance talk used by clients. He proposed a taxonomy involving different *types *of change talk (e.g., desire, reason, ability, commitment, need and readiness), mapping out the *moment *during a therapeutic session when such change talk was expressed as well as its *rate*. Analyses pointed out that an increasing use of commitment change talk throughout a MI session, with no drops toward the end of the session, was predictive of abstinence during the subsequent year [52]. The trajectory and type of change talk that was most predictive of abstinence was not a priori hypothesized, thus suggesting that the mechanism underlying change was also discovered. Subsequent work then established further evidence for this proposed mechanism of change [50].

### Considering the Potential for Systematic Integration between MI and SDT

Most recently, Miller and Rose [3] argued that it is time to begin to move toward a *theory *of motivational interviewing and thus suggested, at least indirectly, that MI can move beyond its bottom-up origins to extend beyond a practice-driven model. Their call for a theory of MI was justified by the fact that both the effectiveness of MI as a clinical method and the mechanisms underlying its observed positive effects have been established. It is noteworthy that Miller and Rose [3] were proposing an "emerging theory of MI" (p. 527) because many questions still remain unanswered. For instance, as noted by Miller and Rose [3]), "if the client elicitation of change talk is reliably linked to commitment and behavior change, *why *is that so?" (p. 534; original italizing). To address this issue and for MI to become a theory rather than remain a model, we believe it is critical to specify its meta-theoretical basis, to clarify its theoretical (dis)similarity with existing theories, and to clearly identify the underlying psychological mechanism of change.

Throughout its discovery-driven development, MI has been linked to a multitude of theories, including Festinger's [53] cognitive dissonance theory, Bem's [54] self-perception theory, and Roger's [55] client-centered approach. For instance, Miller and Rose [3] identify therapist empathy and the Rogerian-based spirit of MI as one of the active components of MI. Most recently, several scholars [8,9] have argued that SDT and MI can potentially be wed because of (a) their complimentary character and (b) their shared features. Such a marriage would allow for a better understanding of the motivational dynamics among a diversity of clinical populations, such as clients with acute suicidal ideation [56] and partner-abusive men [57]. Yet, in our reading of the MI literature, the founders of MI have not fully acknowledged this potential for systematic integration [3], perhaps because they overlooked the papers that argue for such an integration.

Regarding the *complimentarity *between MI and SDT, the motivational communication techniques practiced within MI can inform SDT practitioners. On the other hand, SDT's focus on basic need satisfaction and the internalization of the reason for change allows one to better understand the processes underlying the effectiveness of MI [8,9]. In line with these claims, Fuemmeler et al. [58] reported that an intervention rooted in MI to increase fruit and vegetable consumption promoted a more autonomous regulation. Similarly, in a randomized trial among counseling clients with type 2 diabetes, Rubak et al. [59] found that clients in their MI based intervention group had more strongly self-endorsed (i.e., internalized) the importance of diabetes self-management behaviors at a one year follow-up compared with the clients from the control group. Both groups were being taught intensive treatment of their diabetes.

Notably, MI researchers have not examined the isolated effects of various MI communication techniques in their controlled clinical trials (but see [60]). Such a deconstruction, which has been done in some SDT laboratory [24] and field studies [61], would be worthwhile for both MI and SDT researchers because it would generate more precise insight in the specific components of a MI intervention that carry the effect on proposed SDT based mediators (i.e., basic need satisfaction), MI based mediators (i.e., change talk) and the targeted behavioral and health outcomes. This approach would help to gain insight in which specific communication techniques energize behavior and, as a result, it might lead to refinement of existing MI techniques, and SDT interventions.

Regarding their *shared theoretical focus*, both MI and SDT can both be at least partially traced back to their humanistic and client-centered underpinnings [6,7,62,63]and [64]. Further, in various writings of Miller and Rollnick [6,7] and Deci and Ryan [1] - the founders of MI and SDT, respectively - the issue of autonomy and volition has been repeatedly emphasized. Yet, we note three differences between the SDT and MI view on autonomy.

A first difference concerns the differentiation between autonomy and independence within SDT [65-68], while it is unclear whether such a differentiation is made within MI. It is important to differentiate autonomy, as defined within SDT, from independence, because several clinicians suggest supporting autonomy could be harmful because they may interpret autonomy as independent decision making [69]. The opposite of independence is dependence in which case clients rely on the advice and help of counselor to resolve their problems. A smoker who decides to and succeeds in quitting smoking by himself functions independently, while the smoker who relies on the help and advice of a doctor displays dependent functioning. However, both the self-quitter as well as the doctor-dependent quitter might be quitting for autonomous and volitional or for controlled and pressuring reasons. Thus, the opposite of autonomy within SDT is not dependence but heteronomy, in which case clients' change behaviors are determined by internally or externally pressuring forces. More generally, this conceptual refinement suggests that it is critical to distinguish the *source *of the decision making process (i.e., deciding by oneself vs. deciding with the help of others) from the *phenomenological experience *underlying the decision making process. Based on previous research in the domains of parenting [67,70] and emerging adulthood [71] and SDT, one can predict that especially the autonomous and controlling reasons underlying one's (in)dependent functioning rather than the independent vs. dependent functioning per se would be predictive of clinical success. Thus, a doctor-dependent quitter who willingly relies on the help of his doctor might be as successful as self-quitter who choose to quit by himself, while controlled self- and doctor-dependent quitters are more likely to relapse over time.

Second, because SDT incorporates autonomy (i.e., volition) as a critical theoretical process in its own right, adherence to externally established behavioral goals is considered an incomplete outcome measure. Volitionally non-adherent [72] participants need to be included as a positive outcome to the volitionally adherent for the effectiveness of an SDT intervention to be assessed [73], while it is unclear whether a similar position is maintained within MI. The argumentation to include autonomous non-adherence as a valuable outcome is consistent with medicine's professional charter [74] and biomedical ethics [75] which include respect for autonomy an equivalent outcome to enhancing wellbeing as an outcome. Thus, since clients have the right to choose to be non-adherent, the outcomes of clinical encounters are positive if the clinicians have supported clients' autonomous (or informed) decisions to not accept recommended treatments, and lifestyle changes [76].

To achieve the outcome of autonomous disengagement clinicians would need to bring the client into a position where he can take a well-informed decision. To do so, the clinician would collaboratively discuss pros and cons of change and provide information among other things so that the client can, after reflection, endorse the decision to engage either in the recommended behavior change or treatment course or postpone the change to a later moment. For instance, at the end of a doctor visit a smoker might decide autonomously not to pursue change in the coming three months because he anticipates various life stressors that might trigger his smoking behavior. To further ensure that the patient acts autonomously, the doctor would then ask the client if the doctor provided behavioral counseling, interpersonal support, or medications to facilitate the client's perceived competence to successfully manage those stressors without tobacco. If the client still declined setting a quit date, the doctor would ask permission to bring up the issue of quitting during a new visit after this smoking period. By respecting a client for autonomously disengaging from change, it might become more probable that the client does pursue change for autonomous reasons at a later moment [77,38], while at the same time fulfilling the highest tenets of medical professionalism [74] and biomedical ethics [75]. Interestingly, this respect for volitional non-adherence is consistent with MI's principle of rolling with resistance, and may be facilitative of change talk.

A third difference relates to the way this focus on client autonomy has arisen; that is, the importance of client autonomy has been emphasized for different reasons. Whereas the dynamic of autonomy is said to be universally important and is thus completely theoretically anchored within SDT's top-down perspective, the importance placed on client autonomy and volition has primarily grown out of clinical experience within MI's bottom-up approach. That is, client autonomy was perhaps valued as it yielded desired outcomes (e.g., less drop-out; more progression; less relapse), thus constituting a desirable attribute of clients.

In spite of the call to incorporate client autonomy, when defined as volition, as a theoretical principle rather than just a motivating practice in MI [8,9], Miller and Rose [3] did not embrace this call in their most recent theoretical statement of MI. Instead, Miller and Rose emphasized more strongly the importance of change talk as one of the active components that drive the effectiveness of MI. We argue, however, that for a deeper and systematic integration [78] between MI and SDT to take place MI scholars might want to accept the idea that client autonomy, when defined as the experience of volition and psychological freedom, represents a basic human need. The endorsement of the theoretical principle of autonomy would imply that MI scholars fully embrace SDT's organismic-dialectical meta-theory. In the following, we discuss the advantages of considering autonomy as a fundamental human need.

### The Advantages of Recognizing Autonomy as a Fundamental Human Need and a Theoretical Principle

The recognition that autonomy or volition represent a fundamental human need and a theoretical principle (a) allows avoiding an instrumental approach of MI and client autonomy, (b) allows analyzing change talk within MI in a theory-driven way, thereby distinguishing between different types of change talk, and (c) helps to address the question of whether all clients benefit from autonomy support or whether some clients would benefit more from a directive approach based on their personal preference for being directed to recommended behaviors and treatments.

### Avoiding an Instrumental Approach of MI and Autonomy

Over the past decade, MI has attracted a lot of attention among counselors coming from different psychotherapeutic schools, presumably because MI is perceived as a useful approach to increase client adherence. As a result, MI has been combined with various psychotherapeutic (e.g., cognitive-behavioral) approaches [79]. In those cases, a couple of MI sessions serve as an 'add-on' or 'prelude' prior to the provision of a series of counseling sessions that are grounded in a particular psychotherapeutic school.

Such an approach is fully in line with Miller and Rollnick's argument that MI does not represent a psychotherapeutic school in itself and thus can be used in conjunction with various other approaches. Nevertheless, considering MI as a 'prelude' might lead to instrumental approach of MI; that is, MI might sometimes be treated as a means that helps in reaching desired ends (i.e., promoting health outcomes) and increasing client autonomy is the instrumental pathway to do so. When instrumentally approached, MI in general and client autonomy support in particular might get reduced to a set of techniques, perhaps, even manipulations, that one uses to get the client moving in a particular direction. However, Miller and Rollnick [7] strongly argued against such practice, thereby emphasizing that MI does not represent a technique as the mere application of MI techniques while ignoring its underlying spirit does not represent MI. We believe such an instrumental approach of MI and client autonomy will be more easily avoided if one embraces the idea of autonomy as a fundamental basic need that deserves respect in its own right, regardless of the outcomes it might entail. Indeed, a client might sense that a counselor is promoting autonomy with the sole aim of increasing subsequent adherence and, hence, the counselor would likely be experienced as controlling, and not be experienced as autonomy-supportive, or relationship-supportive. Instead, the idea is that the counselor tries to support the client's autonomy as a valuable goal in its own right, whether doing so increases the chance for adherence to the recommended behavior or not. Client autonomy is thus valued as an end, or a outcome, in and of itself. The increase in adherence that results from an autonomy-supportive style would thus not be the focal goal of being autonomy-supportive but it would be a *by-product *that follows from an autonomy-supportive style.

To avoid such an instrumental approach of MI and the concept of autonomy, we believe that it is critical to combine MI with psychotherapeutic approaches that share a similar meta-theoretical foundation. If not, the clinical MI practices in the early sessions might at some points conflict with the clinical practices used during the subsequent sessions. For instance, although client autonomy might be fully respected in the early MI-based sessions, some counselors, when trying to ensure continued adherence during the subsequent sessions, might turn towards rewards or contracting clients, which might compromise the client's autonomy and volition. However, counselors who are working within a psychotherapeutic school (e.g., client-centered psychotherapy) that fully endorses the principle of client autonomy would not rely on autonomy-thwarting practices during the post-MI counseling sessions and, hence, no confusion would arise for the clients between the initial MI and subsequent sessions grounded in another approach.

### A Qualitative Analysis of Change Talk

As noted, the identification of mediating mechanisms accounting for the positive effects of MI largely constituted a discovery trip rather than being an a priori theorized endeavor. For instance, it was discovered that only particular types of change talk (i.e., commitment language) were predictive of abstinence [52]. The examination of this change talk can, however, also occur in a more theory-driven fashion. Several of the change talk categories (i.e., commitment, desire, need, readiness, and reasons) as distinguished by Amrheim et al. [52] could be differently approached from concept of autonomy or volition as defined within SDT. For each of these categories, Amrheim et al. [52] assigned a greater value if the client more strongly verbally expressed his commitment, desire, need or readiness to change; thus, a higher value reflected a higher level of motivation to pursue change. For instance, a verbal change expression as 'I won't be using', 'I need to stop' and 'I'm ready to do this' were assigned a high commitment, need and readiness score.

Although such change talk might be more easily expressed by autonomously motivated clients it does not need to be the case. From the SDT perspective, change talk expressions can also be instigated by controlling forces and, hence, change talk does not by definition reflect a willing pursuit of change. For instance, a smoker who feels guilt for not taking care of his health might well say that 'I won't be using in the future' or that 'I need to change my smoking behavior'. Also, autonomously and controlled motivated clients might express a different *kind *of change talk, with controlling individuals using more pressuring language and being more rigidly focused on symptom change (e.g., "I have to lose weight") and autonomous clients using more autonomy-supportive change talk and being more focused on the process of personality and life style changes (e.g., "It would be good if I could exercise more"; see [80,81]). Further, autonomous and controlled clients might verbalize the same change talk in a *different way*. For instance, the emotional tone underlying the change talk might be different, with controlled, relative to autonomously, motivated clients expressing less enthusiasm, more tension and more negative emotions. Also, whereas controlled motivated clients' change talk might be fluctuating throughout the session, being indicative of self-doubt and uncertainty, the change talk of autonomously motivated clients might be less susceptible to momentary changes.

In short, we argue that the concept of autonomy or volition vs. control or coercion allows for a qualitative analysis of change talk, thereby examining different categories of change talk, different ways of expressing change talk and formulating predictions regarding the variability vs. stability of change talk within and across sessions. If future research demonstrates that it is not the quantity of change talk by itself but rather the type of change talk, the emotional tone and the variability of change talk that predicts outcomes (e.g., abstinence and well being), it might suggest that change talk by itself just represents a *surface manifestation *of a more fundamental underlying process, that is, a change towards a more integrated and volitional model of functioning.

### Do Some Clients Benefit from Directive Counseling?

One of the issues often discussed within MI is whether some clients might benefit from a directive rather than an autonomy-supportive counseling style, because the latter style does not align with their preference for directiveness [82]. Given that autonomy represents an inborn, universal need, it is maintained within SDT that all clients will benefit from autonomy-supportive counseling. The question whether some clients would benefit from directive counseling is not easily addressed from the SDT perspective, because this term carries multiple meanings. Spelling out these different meanings might help to shed light on an issue that has attracted considerable attention among MI scholars.

First, directive counseling can refer to coercive counseling, in which case clients are pushed to adopt a particular way of thinking, behaving or feeling. In this case, the term directive counseling is contrasted with autonomy-supportive counseling, which has the aim of supporting clients' volition and willingness to pursue change, thereby satisfying the basic need for autonomy. Second, the term directive can also mean providing structure. Structuring clinicians will provide information and help, educate and teach skills, thereby supporting the client's need for competence. When conceptualized in this way, the opposite of directiveness is not autonomy support, but a laissez-faire style, where the client is abandoned to himself to make it alone.

Giving this double meaning of directive counseling, the question whether some clients might benefit from directive counseling can be reconsidered. When yielding the meaning of being coercive and controlling, both MI and SDT would argue that no client will benefit from being pushed around. On the basis of SDT, a theoretical account could be provided for this argument because it is maintained that all clients possess a basic need for autonomy and volition, which gets thwarted when counselors are controlling.

When directive means providing structure with an active involvement of client and practitoner, SDT maintains that clients might benefit from this structure if it is provided in an autonomy-supportive rather than a coercive way [83]. Thus, the style of providing information, teaching skills, and educating will determine whether the provision of information, education and learning of skills is experienced as supportive or as controlling. When clients feel controlled, providing the information likely prompts a defensive reaction. An example might illustrate this. A dietician could begin educating an obese person about which high calorie foods to avoid before asking what the client knows or if he wants to change. When the provided information is redundant or undesired, it is more likely that the client will feel externally pressured by the dietician to change. Indeed, the structure provided in this manner is likely to inhibit internalization of the reason for change and may elicit overt resistance. The chance then that the client will deeply process the offered information and integrate this information into his lifestyle is low. This attempt at competence-support without autonomy support is not expected to yield sustained motivation for change because it is experienced as control. Instead, when the client expresses a preference for information, and wants the clinician to tell him what to do to achieve his goals, the provided information and competence building will be welcomed and, hence, better processed and more efficiently applied in practice.

This suggests that clinicians would do well to first explore whether there is a request for information, skill teaching or help. This will require empathy and sensitivity from the side of the clinician, who tries to fully elicit and acknowledge the client's perspective. Collaboratively, they explore first whether there is willingness to pursue change and, if so, what the client needs to make change happen. Thus, the initial autonomy and relatedness support provided determine whether the counselor's recommendations and efforts at skill-building and information provision will be experienced as need-supportive or need-frustrating.

It is striking to us that a similar view is maintained within MI. Specifically, Resnicow (this issue) suggested providing information using an 'elicit-provide-elicit' framework. The counselor first *elicits *the person's understanding and desire for information, then *provides *new information in a neutral manner, followed by *eliciting *what this information might mean for the client, using a question such as, "What does this mean to you" or "How do you make sense of all this?". MI practitioners avoid trying to persuade clients with "pre-digested" health messages and instead allow clients to process information and find what is personally relevant for them. This approach allows for competence-building, while at the same type respecting client autonomy, as the client is asked how much information he might desire and what he thinks of newly provided information.

Thus, SDT and MI converge on how clinicians would handle the request for information and recommendations regarding their health, although they use different vocabulary for doing so. Also, MI is primarily focused on how this can be concretely done (e.g., 'elicit-provide-elicit practice'), while SDT scholars provide an account of the processes (i.e., basic need satisfaction) underlying these motivational practices. Thus, it might be worthwhile for MI scholars to recognize the multiple meanings that the term 'directiveness' carries and to endorse the concept of autonomy as a theoretical principle. Doing so would allow researchers in both camps to generate and test more precise hypotheses that will inform which counseling practices are more motivating for clients, which result in greater well being for clients, and why these outcomes occurred.

## Conclusion

Both MI and SDT have received increasing attention in the literature, with MI being rapidly spread in various health care domains and SDT being tested in health care and in a number of additional domains, such as work, education, sports and exercise, and ecology. We believe that MI and SDT, although characterized by different starting points and undergoing a different development (i.e., bottom-up vs. top-down), can potentially be integrated with continued careful consideration of the underlying concepts and their appropriate empirical validation. For such systematic integration to occur, though, we maintain that autonomy, defined as the clients' experience of a sense of volition and psychological freedom, needs to be considered as a fundamental human need and theoretical process, and as a clinical endpoint by itself. Establishing autonomy as a basic human need, which the satisfaction of, together with competence and relatedness, energizes human behavior, will inform clinicians' behavior and the structure of interventions that will enhance integration, wellness, and growth of the human organism.

## Competing interests

The authors declare that they have no competing interests.

## Authors' contributions

MV took the lead in writing the manuscript and GW and KR both provided useful comments to improve the contribution. All authors read and approved the final manuscript.

## References

[B1] Deci EL Ryan RM The 'what' and 'why' of goal pursuits: Human needs and the self-determination of behavior Psychological Inquiry 2000 11 227 268 10.1207/S15327965PLI1104_01

[B2] Ryan RM Deci EL Self-determination theory and the facilitation of intrinsic motivation, social development, and well-being American Psychologist 2000 55 68 78 11392867 10.1037//0003-066x.55.1.68

[B3] Miller WR Rose GS Toward a theory of motivational interviewing American Psychologist 2009 64 527 537 19739882 10.1037/a0016830 PMC2759607

[B4] Maciosek MV Edwards NM Coffield AB Priorities among effective clinical preventive services American Journal of Preventive Medecine 2006 31 90 6 10.1016/j.amepre.2006.03.011 16777547

[B5] Rothman AJ "Is there nothing more practical than a good theory?": Why innovations and advances in health behavior change will arise if interventions are used to test and refine theory International Journal of Behavioral Nutrition and Physical Activity 2004 1 11 10.1186/1479-5868-1-11 15279674 PMC514573

[B6] Miller WR Rollnick S Motivational interviewing 2002 New York: Guilford

[B7] Miller WR Rollnick S Ten things that motivational interviewing is not Behavioural and Cognitive Psychotherapy 2009 37 129 140 10.1017/S1352465809005128 19364414

[B8] Markland D Ryan RM Tobin VJ Rollnick S Motivational interviewing and self-determination theory Journal of Social and Clinical Psychology 2005 24 811 831 10.1521/jscp.2005.24.6.811

[B9] Vansteenkiste M Sheldon KM 'There is nothing more practical than a good theory': Integrating motivational interviewing and self-determination theory British Journal of Clinical Psychology 2006 45 63 82 10.1348/014466505X34192 16480567

[B10] Lewin K Field theory in social science: selected theoretical papers by Kurt Lewin 1952 London: Tavistock Publications

[B11] Deci EL Vansteenkiste M Self-determination theory and basic need satisfaction: Understanding human development in positive psychology Ricerche di Psichologia 2004 27 17 34

[B12] Vansteenkiste M Niemiec C Soenens B Urdan T, Karabenick S The development of the five mini-theories of self-determination theory: An historical overview, emerging trends, and future directions Advances in Motivation and Achievement: The Decade Ahead 2010 16 UK: Emerald Publishing 105 166

[B13] Deci EL Intrinsic motivation 1975 New York: Plenum Publishing Co

[B14] Ryan RM Deci EL A self-determination approach to psychotherapy: The motivational basis for effective change Canadian Psychology 2008 49 186 193

[B15] Patall EA Cooper H Robinson C The effects of choice on intrinsic motivation and related outcomes: A meta-analysis of research findings Psychological Bulletin 2008 134 270 300 18298272 10.1037/0033-2909.134.2.270

[B16] Zuckerman M Porac JF Lathin D Smith R Deci EL On the importance of self-determination for intrinsically motivated behavior Personality and Social Psychology Bulletin 1978 4 443 446 10.1177/014616727800400317

[B17] Clark NM Janz NK Dodge JA The effect of client choice of intervention on health outcomes Contemporary Clinical Trials 2008 29 679 686 10.1016/j.cct.2008.04.002 18515187 PMC2577598

[B18] Vandereycken W Vansteenkiste M "Let eating disorder clients decide!": Providing choice might reduce early drop-out in inclient treatment European Eating Disorder Review 2009 17 177 183 10.1002/erv.917 19306300

[B19] Philippe FL Vallerand RJ Actual environments do affect motivation and psychological adjustment: A test of self-determination theory in a natural setting Motivation & Emotion 2008 32 81 89 10.1007/s11031-008-9087-z22396626

[B20] Mouratidis A Vansteenkiste M Lens W Sideridis G Class-to-class variation in vitality and intrinsic motivation in physical education as a function of the synergistic interaction between need-supportive teaching and pupils' motivational orientations Journal of Educational Psychology 2011 in press

[B21] Ward J Wilkinson C Graser SV Prusak KA Effects of choice on student motivation and physical activity behavior in physical education Journal of Teaching in Physical Education 2008 27 385 398

[B22] Jang H Supporting students' motivation, engagement, and learning during an uninteresting activity Journal of Educational Psychology 2008 100 798 811

[B23] Koestner R Ryan RM Bernieri F Holt K Setting limits on children's behavior: The differential effects of controlling versus informational styles on children's intrinsic motivation and creativity Journal of Personality 1984 54 233 248

[B24] Deci EL Eghrari H Patrick BC Leone DR Facilitating internalization: The self-determination theory perspective Journal of Personality 1994 62 119 142 10.1111/j.1467-6494.1994.tb00797.x 8169757

[B25] Ryan RM Control and information in the intrapersonal sphere: An extension of cognitive evaluation theory Journal of Personality and Social Psychology 1982 43 450 461

[B26] Vansteenkiste M Simons J Lens W Sheldon KM Deci EL Motivating learning, performance, and persistence: The synergistic role of intrinsic goals and autonomy-support Journal of Personality and Social Psychology 2004 87 246 260 15301630 10.1037/0022-3514.87.2.246

[B27] Chatzisarantis NLD Hagger MS Effects of an intervention based on Self-Determination Theory on self-reported leisure-time physical activity participation Psychology and Health 2009 24 29 48 10.1080/08870440701809533 20186638

[B28] Williams GC McGregor H Sharp D Levesque CS Kouides RW Ryan RM Deci EL Testing a self-determination theory intervention for motivating tobacco cessation: Supporting autonomy and competence in a clinical trial Health Psychology 2006 25 91 101 16448302 10.1037/0278-6133.25.1.91

[B29] Halvari AEM Halvari H Motivational predictors of change in oral health: An experimental test of self-determination theory Motivation and Emotion 2006 30 295 306

[B30] Silva MN Vieira PN Coutinho SR Minderico CS Matos MG Sardinha LS Teixeira PJ Using self-determination theory to promote physical activity and weight control: a randomized controlled trial in women Journal of Behavioral Medicine 2010 33 110 122 10.1007/s10865-009-9239-y 20012179

[B31] Ryan RM Patrick H Deci EL Williams GC Facilitating health behaviour change and its maintenance: Interventions based on self-determination theory The European Health Psychologist 2008 10 2 5

[B32] Sheldon KM Filak V Manipulating autonomy, competence, and relatedness support in a game-learning context: New evidence that all three needs matter British Journal of Social Psychology 2008 47 267 283 10.1348/014466607X238797 17761025

[B33] Moller AC Deci EL Ryan RM Choice and ego-depletion: The moderating role of autonomy Personality and Social Psychology Bulletin 2006 32 1024 1036 10.1177/0146167206288008 16861307

[B34] Iyengar SS Lepper MR When choice is demotivating: Can one desire too much of a good thing? Journal of Personality and Social Psychology 2000 79 995 1006 11138768 10.1037//0022-3514.79.6.995

[B35] Ogden CL Yanovski SZ Carroll MD Flegal KM The epidemiology of obesity Gastroenterology 2007 132 2087 2102 10.1053/j.gastro.2007.03.052 17498505

[B36] Ricciardelli LA McCabe P Dietery restraint and negative affect as mediators of body dissatisfaction and bulimic behavior in adolescent girls and boys Behavior Research and Therapy 2001 39 1317 1328 10.1016/S0005-7967(00)00097-8 11686266

[B37] Hoek HW van Hoeken D Review of the prevalence and incidence of eating disorders International Journal of Eating Disorders 2003 34 383 396 10.1002/eat.10222 14566926

[B38] Fiore MC Jaen CR Baker TB Bailey WC Benowitz NL Curry SJ Treating Tobacco Use and Dependence: 2008 2008 Update: U.S. Department of Health and Human Services

[B39] Miller WR Baca LM Two-year follow-up of bibliotherapy and therapist-dircted controlled drinking training for problem drinkers Behavior Therapy 1983 14 441 448 10.1016/S0005-7894(83)80107-5

[B40] Miller WR Taylor CA West JC Focused versus broad spectrum behavior therapy for problem drinkers Journal of Consulting and Clinical Psychology 1980 48 509 601 10.1037//0022-006x.48.5.590 7410657

[B41] Miller WR Motivational interviewing with problem drinkers Behavioural Psychotherapy 1983 11 147 172 10.1017/S0141347300006583

[B42] Burke BL Arkowitz H Menchola M The efficacy of motivational interviewing: A meta-analysis of controlled clinical trials Journal of Consulting and Clinical Psychology 2003 71 843 61 14516234 10.1037/0022-006X.71.5.843

[B43] Hettema J Steele J Miller WR Motivational interviewing Annual Review of Clinical Psychology 2005 1 91 111 10.1146/annurev.clinpsy.1.102803.143833 17716083

[B44] Lundahl BW Tollefson D Kunz C Brownell C Burke B Meta-analysis of motivational interviewing: Twenty five years of research Research on Social Work Practice 2010 20 137 160 10.1177/1049731509347850

[B45] Rubak S Sandboek A Lauritzen T Christensen B Motivational interviewing: A systematic review and meta-analysis British Journal of General Practice 2005 81 305 312 PMC1463134 15826439

[B46] Vasilaki EI Hosier SG Cox WM The efficacy of motivational interviewing as a brief intervention for excessive drinking: A meta-analytic review Alcohol and Alcoholism 2006 41 328 335 10.1093/alcalc/agl016 16547122

[B47] Brenman L Walkley J Fraser SF Greenway K Wilks R Motivational interviewing and cognitive behavior therapy in the treatment of adolescent overweight and obesity: Study design and methodology Contemporary Clinical Trial 2008 29 359 375 10.1016/j.cct.2007.09.001 17950046

[B48] Dean HY Touyz SW Rieger E Thornton CE Group motivational enhancement therapy as an adjunct to inpatient treatment for eating disorders: A preliminary study European Eating Disorders Review 2008 16 256 267 10.1002/erv.851 18059067

[B49] Moyers TB Martin T Therapist influence on client language during motivational interviewing sessions: Support for a potential causal mechanism Journal of Substance Abuse Treatment 2006 30 245 251 10.1016/j.jsat.2005.12.003 16616169

[B50] Moyers TB Martin T Christopher PJ Houck JM Tonigan JS Amrhein PC Client language as a mediator of motivational interviewing efficacy: Where is the evidence? Alcoholism: Clinical and Experimental Research 2007 31 40 47 10.1111/j.1530-0277.2007.00492.x 17880345

[B51] Miller WR Yahne CE Tonigan JS Motivational interviewing in drug abuse services: A randomized clinical trial Journal of Consulting and Clinical Psychology 2003 71 754 763 12924680 10.1037/0022-006x.71.4.754

[B52] Amrhein PC Miller WR Yahne CE Paler M Fulcher L Client commitment language during motivational interviewing predicts drug use outcomes Journal of Consulting and Clinical Psychology 2001 71 862 878 10.1037/0022-006X.71.5.862 14516235

[B53] Festinger L A theory of cognitive dissonance 1957 Standford, CA: Standford University Press

[B54] Bem DJ Self-perception: An alternative interpretation of cognitive dissonance phenomena Psychological Review 1967 74 183 200 5342882 10.1037/h0024835

[B55] Rogers CR Client-centered therapy 1951 Boston: Houghton-Mifflin

[B56] Britton PC Williams GC Conner KR Self-determination theory, motivational interviewing, and the treatment of clients with acute suicidal ideation Journal of Clinical Psychology 2008 64 52 66 10.1002/jclp.20430 18161032

[B57] Neighbors C Roffman RA Bmilinyi LF Edleson JL Self determination theory and motivational interviewing: Complementary models to elicit voluntary engagement by partner-abusive men American Journal of Family Therapy 2008 36 126 136 10.1080/01926180701236142 PMC3351133 22593609

[B58] Fuemmeler BF Masse LC Yaroch AL Resnicow K Campbell MK Carr C Psychosocial mediation of fruit and vegetable consumption in the Body and Soul effectiveness trial Health Psychology 2006 25 474 83 16846322 10.1037/0278-6133.25.4.474

[B59] Rubak S Sandboek A Lauritzen T Borch-Johnsen K Christensen B General practitioners trained in motivational interviewing can positively affect the attitude to behaviour change in people with type 2 diabetes Scandinavian Journal of Primary Health Care 2009 27 172 179 10.1080/02813430903072876 19565411 PMC3413190

[B60] Glynn LH Moyers TB Chasing change talk: The clinician's role in evoking client language about change Journal of Substance Abuse Treatmen 2010 39 65 70 10.1016/j.jsat.2010.03.012 20418049

[B61] Reeve J Bolt E Cai Y Autonomy-supportive teachers: How they teach and motivate students Journal of Educational Psychology 1999 91 537 548

[B62] Patterson PG Joseph S Person-centered personality theory: Support from self-determination theory and positive psychology Journal of Humanistic Psychology 2007 47 117 139 10.1177/0022167806293008

[B63] Sheldon KM Kasser T Goals, congruence, and positive well-being: New empirical validation for humanistic ideas Journal of Humanistic Psychology 2001 41 30 50 10.1177/0022167801411004

[B64] Williams GC Frankel RM Campbell TL Deci EL Research on relationship-centered care and health-care outcomes from the Rochester Biopsychosocial Program: A self-determination theory integration Families, Systems & Health 2000 18 79 9022396869

[B65] Chirkov V Ryan RM Kim Y Kaplan U Differentiating autonomy from individualism and independence: A self-determination theory perspective on internalization of cultural orientations and well-being Journal of Personality and Social Psychology 2003 84 97 110 12518973

[B66] Ryan RM Jacobs J Agency and organization: Intrinsic motivation, autonomy and the self in psychological development Nebraska symposium on motivation: Developmental perspectives on motivation 1993 40 Lincoln, NE: University Of Nebraska Press 1 56 1340519

[B67] Soenens B Vansteenkiste M Lens W Luyckx K Beyers W Goossens L Ryan RM Conceptualizing parental autonomy support: Adolescent perceptions of promoting independence versus promoting volitional functioning Developmental Psychology 2007 43 633 646 17484576 10.1037/0012-1649.43.3.633

[B68] Vansteenkiste M Zhou M Lens W Soenens B Experiences of autonomy and control among Chinese learners: Vitalizing or immobilizing? Journal of Educational Psychology 2005 97 468 483

[B69] Epstein RM Korones DN Quill TE Withholding information from patients - when less is more New England Journal of Medicine 2010 362 380 381 10.1056/NEJMp0911835 20130252

[B70] Soenens B Vansteenkiste M Sierens E How are parental psychological control and autonomy-support related? Naturally occurring profiles of psychological control and two types of autonomy-support Journal of Marriage and the Family 2009 71 187 202 10.1111/j.1741-3737.2008.00589.x

[B71] Kins E Beyers W Soenens B Vansteenkiste M Patterns of home leaving subjective well-being in emerging adulthood: The role of motivational processes and parental autonomy support Developmental Psychology 2009 45 1416 1429 19702402 10.1037/a0015580

[B72] Vansteenkiste M Lens W Dewitte S De Witte H Deci EL The "why" and "why not" of job search behavior: Their relation to searching, unemployment experience and well-being European Journal of Social Psychology 2004 34 345 363 10.1002/ejsp.202

[B73] Ryan RM Lynch MF Vansteenkiste M Deci EL Motivation and autonomy in counseling, psychotherapy, and behavior change: A look at theory and practice Counseling Psychologist 2011 39 193 260 10.1177/0011000009359313

[B74] ABIM Foundation, ACP-ASIM Foundation, European Federation of Internal Medicine Medical professionalism in the new millennium: A physician charter Annals of Internal Medicine 2002 136 243 6 11827500 10.7326/0003-4819-136-3-200202050-00012

[B75] Beauchamp TL Childress JF Principles of biomedical ethics 2009 New York: Oxford University Press

[B76] Woolf SH Chan ECY Harris R Sheridan SL Braddock CH Kaplan RM Promoting informed choice: Transforming health care to dispense knowledge for decision making Annals of Internal Medicine 2005 143 293 300 16103473 10.7326/0003-4819-143-4-200508160-00010

[B77] Williams GC Quill TE Deci EL Ryan RM The facts concerning the recent carnival of smoking in Connecticut (and elsewhere) Annals of Internal Medicine 1991 115 59 63 2048862 10.7326/0003-4819-115-1-59

[B78] Patterson CH Foundations for a systematic eclectic psychotherapy Psychotherapy 1989 26 427 435

[B79] Buckner JD Schmidt NB A randomized pilot study of motivation enhancement therapy to increase utilization of cognitive-behavioral therapy for social anxiety Behaviour Research and Therapy 2009 47 710 715 10.1016/j.brat.2009.04.009 19467647

[B80] Conroy DE Coatsworth JD Coaching behaviors asociated with changes in fear of failure: Changes in self-talk and need satisfaction as potential mechansims Journal of Personality 2007 75 383 419 10.1111/j.1467-6494.2006.00443.x 17359243

[B81] Oliver EJ Markland D Hardy J Petherick CM The effects of autonomy-supportive versus controlling environments on self-talk Motivation & Emotion 2008 32 200 212 10.1007/s11031-008-9097-x22396626

[B82] K Resnicow R Davis G Zhang J Konkel V Strecher A Shaikh D Tolsma J Calvi G Alexander J Anderson C Wiese Tailoring a fruit and vegetable intervention on novel motivational constructs: results of a randomized study Annual Behavioral Medicine 2008 35 159 169 10.1007/s12160-008-9028-9 18401673

[B83] Sierens E Vansteenkiste M Goossens L Soenens B Dochy F The synergistic relationship of perceived autonomy-support and structure in the prediction of self-regulated learning British Journal of Educational Psychology 2009 79 57 68 10.1348/000709908X304398 18466671

